# Iron-Dependent Mitochondrial Dysfunction Contributes to the Pathogenesis of Pulmonary Fibrosis

**DOI:** 10.3389/fphar.2021.643980

**Published:** 2022-01-04

**Authors:** Mai Takahashi, Kenji Mizumura, Yasuhiro Gon, Tetsuo Shimizu, Yutaka Kozu, Sotaro Shikano, Yuko Iida, Mari Hikichi, Shinichi Okamoto, Kota Tsuya, Asami Fukuda, Shiho Yamada, Kaori Soda, Shu Hashimoto, Shuichiro Maruoka

**Affiliations:** Division of Respiratory Medicine, Department of Internal Medicine, Nihon University School of Medicine, Tokyo, Japan

**Keywords:** pulmonary fibrosis, iron metabolism, mitochondrial dysfunction, cigarette smoke, reactive oxygen species

## Abstract

Although the pathogenesis of pulmonary fibrosis remains unclear, it is known to involve epithelial injury and epithelial-mesenchymal transformation (EMT) as a consequence of cigarette smoke (CS) exposure. Moreover, smoking deposits iron in the mitochondria of alveolar epithelial cells. Iron overload in mitochondria causes the Fenton reaction, leading to reactive oxygen species (ROS) production, and ROS leakage from the mitochondria induces cell injury and inflammation in the lungs. Nevertheless, the mechanisms underlying iron metabolism and pulmonary fibrosis are yet to be elucidated. In this study, we aimed to determine whether iron metabolism and mitochondrial dysfunction are involved in the pathogenesis of pulmonary fibrosis. We demonstrated that administration of the iron chelator deferoxamine (DFO) reduced CS-induced pulmonary epithelial cell death, mitochondrial ROS production, and mitochondrial DNA release. Notably, CS-induced cell death was reduced by the administration of an inhibitor targeting ferroptosis, a unique iron-dependent form of non-apoptotic cell death. Transforming growth factor-β-induced EMT of pulmonary epithelial cells was also reduced by DFO. The preservation of mitochondrial function reduced Transforming growth factor-β-induced EMT. Furthermore, transbronchial iron chelation ameliorated bleomycin-induced pulmonary fibrosis and leukocyte migration in a murine model. Our findings indicate that iron metabolism and mitochondrial dysfunction are involved in the pathogenesis of pulmonary fibrosis. Thus, they may be leveraged as new therapeutic targets for pulmonary fibrosis.

## Introduction

Idiopathic pulmonary fibrosis (IPF) is a life-threatening lung disease that predominantly affects older patients and has a death rate worse than that of many cancers (Lederer and Martinez, 2018). The major risk factors for IPF are tobacco smoke and aging (Lederer and Martinez, 2018; Raghu et al., 2018). Moreover, because inflammation was believed to be the main mechanism underlying IPF pathogenesis, steroid therapy has long been used to treat it. However, recent studies have revealed that recurrent injury and aberrant repair of alveolar cells leading to the deposition of interstitial fibrosis are more suitable explanations of the mechanism of IPF pathogenesis (Raghu et al., 2015). Nintedanib and pirfenidone, two antifibrotic agents that attenuate disease progression, have been approved for the treatment of IPF; however, there is a lack of therapeutic options that can alter the course of this disease (Richeldi et al., 2017; Lederer and Martinez, 2018). Although the mechanisms of lung fibrosis in patients with IPF remain to be fully explained, a recent study suggested the epithelial–mesenchymal transition (EMT) during the healing process as a possible mechanism (Cloonan et al., 2016).

Iron metabolism plays a crucial role in the pathogenesis of pulmonary diseases (Ghio et al., 2008; Cloonan et al., 2016; Cloonan et al., 2017). Tobacco contains 440–1,150 mg iron/g, with ∼0.1% of this iron entering mainstream smoke (Mussalo-Rauhamaa et al., 1986). Although this quantity is not significant, specific host sources of iron (e.g., the cellular labile iron pool) can be bound by particulate surface matter after its deposition in the lung (Ghio et al., 2008). Such complexation of host iron by cigarette smoke (CS) particles likely alters iron homeostasis in the lung. In fact, iron is known to accumulate in the lungs of cigarette smokers (Ghio et al., 2008). Additionally, CS-exposed mice show higher non-heme iron in inflated lung sections and whole-lung homogenates as compared with room-air-exposed mice (Cloonan et al., 2016). Furthermore, iron overload causes a Fenton reaction, which leads to hydroxyl radical production at the cellular level and induces cell injury and death, fibrosis, and inflammation (Bogdan et al., 2016). Specifically, recent studies have uncovered the existence of a unique iron-dependent form of non-apoptotic cell death termed “ferroptosis,” which is characterized by the accumulation of lipid peroxides (Dixon et al., 2012). Aging and cellular senescence are closely associated with the pathogenesis of IPF (King et al., 2011), and an increased level of ferritin, the cellular iron-storage protein, reflects ongoing aging-associated inflammation (Cankurtaran et al., 2012). A previous study reported that enrichment of senescent cells accompanied iron accumulation (Masaldan et al., 2018), and that serum ferritin levels correlate with the severity of anti-MDA5 antibody-associated acute interstitial lung disease (Gono et al., 2011). Moreover, another study showed that iron chelation through inhaled DFO ameliorated lung fibrosis *in vivo* (Ali et al., 2020). However, the specific mechanisms by which iron metabolism can promote pulmonary fibrosis remain incompletely understood. Therefore, we intend to investigate the possible relationship between iron metabolism and cellular regulatory mechanisms in the pathogenesis of pulmonary fibrosis.

Mitochondria produce energy via oxidative phosphorylation and are essential for regulating critical cellular processes, such as cell death and inflammation. We previously reported that mitophagy, the autophagy-dependent elimination of mitochondria, regulates a CS-induced genetically programmed and regulated form of necrosis termed “necroptosis” in pulmonary epithelial cells (Mizumura et al., 2014). CS causes significant mitochondrial depolarization and induces mitophagy in lung epithelial cells, and we demonstrated that the mitophagy inhibitor mdivi-1 protected against CS-induced cell death by inhibiting the necroptosis pathway. Thus, our results suggested that CS-activated mitophagy may alter mitochondrial membrane integrity and induce mitophagy and necroptosis in pulmonary epithelial cells. Furthermore, we had previously revealed that PTEN-induced kinase 1 is the key molecule that mediates CS-induced mitochondrial dysfunction and mitophagy in pulmonary epithelial cells (Mizumura et al., 2014). Subsequently, Bueno et al. (2015) also showed that PTEN-induced kinase 1 deficiency impaired mitochondrial homeostasis and promotes lung fibrosis. These observations led us to hypothesize that CS-induced mitochondrial dysfunction may be involved in the pathogenesis of IPF.

Accumulating evidence also suggests that mitochondrial DNA (mtDNA) acts as a damage-associated molecular pattern that can drive molecular processes that lead to inflammatory responses (Zhang et al., 2010a). Investigators have recently reported that cellular mtDNA levels are decreased (Pyle et al., 2010; Ryu et al., 2017) and circulating cell-free mtDNA levels are increased in response to stimuli, such as IPF (Ryu et al., 2017), trauma (Zhang et al., 2010a), and infection (Cossarizza et al., 2011; Sursal et al., 2013). Moreover, extracellular mtDNA levels can be predictive of mortality in IPF (Ryu et al., 2017) and intensive care unit patients (Nakahira et al., 2013).

Mitochondria are a site of Fe–S cluster biosynthesis and heme synthesis. Therefore, mitochondria are closely associated with iron metabolism. A previous study showed that mitochondrial iron chelation alleviated CS-induced pulmonary inflammation and CS-associated lung injury in mice (Cloonan et al., 2016), and a recent report indicated that mitochondrial fusion is associated with lung alveolar type 2 epithelial cell injury and subsequent fibrotic remodeling in the lungs (Chung et al., 2019). These observations along with our previous studies implicating impaired mitochondrial integrity as a pro-pathogenic mediator in chronic obstructive pulmonary disease (Mizumura et al., 2014) led us to hypothesize that the mitochondrial iron axis is involved in the pathogenesis of pulmonary fibrosis. In this study, we determined the involvement of iron metabolism and mitochondrial dysfunction in the pathogenesis of pulmonary fibrosis.

## Materials and Methods

### Reagents

The antibodies used were as follows: rabbit antibody against human E-cadherin (24E10; Cell Signaling Technology, Danvers, MA, United States); rabbit antibody against human N-cadherin (GTX127345; GENETEX, Inc., Irvine, CA, United States); rabbit antibody against human vimentin (EPR3776; Abcam, Cambridge, United Kingdom); rabbit antibody against human β-actin (4967; Bioss Antibodies, Woburn, MA, United States); and HRP-conjugated secondary antibody against rabbit IgG (7074; Cell Signaling Technology). The inhibitors used were as follows: deferoxamine (DFO; D9533; Sigma-Aldrich, St. Louis, MO, United States), ferrostatin-1 (SML0583; Sigma-Aldrich), and MitoQ (B1309; Biovision, Milpitas, CA, United States).

### Cell Culture

Human lung bronchial epithelial BEAS-2B cells were obtained from the American Type Culture Collection (Manassas, VA, United States) and maintained in DMEM containing 10% FBS and 1% gentamicin. Alveolar epithelial A549 cells were obtained from the JCRB Cell Bank (National Institutes of Biomedical Innovation, Health and Nutrition, Ibaraki City, Japan) and maintained in DMEM containing 10% FBS and 1% penicillin-streptomycin.

### Cigarette Smoke Extract Treatment

A paper cigarette (Peace; Japan Tobacco Inc., Tokyo, Japan; tar, 28 mg; nicotine, 2.3 mg) with the filter removed was ignited and sucked using a peristaltic pump; the tobacco smoke produced was bubbled into cell culture medium (i.e., DMEM). Each cigarette was smoked in 6 min, and 10 ml of cell culture solution was used for each cigarette. The solution was filtered through a sterilization filter (Stericup [250 ml]; Durapore PVDF membrane [0.45 μm]; Millipore, Burlington, MA, United States) and used as the CSE. For cell death experiments, 25% CSE was used, while 5% CSE was used to confirm whether a change in mitochondrial function occurred at a concentration at which cell death did not occur.

### Flow Cytometry

A LIVE/DEAD viability/cytotoxicity kit (L3224; Molecular Probes, Eugene, OR, United States) was used to discriminate between live and dead cells. The cells were simultaneously stained with green fluorescent calcein-AM to detect intracellular esterase activity and with red fluorescent ethidium homodimer-1 to detect the loss of plasma membrane integrity. Data were acquired using a FACSCalibur cytometer (BD Biosciences, Franklin Lakes, NJ, United States). To evaluate lipid peroxidation, the cells were stained with the Image-iT lipid peroxidation kit for live cell analysis (C10445; Thermo Fisher Scientific, Waltham, MA, United States). To assess the functional mitochondrial pool, cells were stained with 100 nM tetramethylrhodamine ethyl ester (ab113852; Abcam) for 20 min at 37°C and then treated with CSE. Cellular mitochondrial reactive oxygen species (mtROS) levels were measured using MitoSOX (M36008 [2.5 μM]; Thermo Fisher Scientific) staining for 10 min at 37°C. Fluorescence was measured and analyzed using the Kaluza for Gallios software (Beckman Coulter, Brea, CA, United States).

### Preparation and Quantification of Mitochondrial Deoxyribonucleic Acid

The cell culture solution obtained from BEAS-2B cells was centrifuged at 100 g for 1 min, and the supernatant was collected and further centrifuged at 20,000 g for 3 min. After removing the supernatant, the precipitate was suspended in TE buffer (10 mM Tris-HCl, 0.1 mM EDTA, pH 8.0) and heat-treated at 95°C for 10 min. After centrifugation at 20,000 g for 1 min, the supernatant was used as the sample. The amount of mtDNA was quantified using quantitative real-time polymerase chain reaction (TaQman gene expression master mix, 4369016; Thermo Fisher Scientific). The primer used for mtDNA was MT-CO1 (10025636; Bio-Rad Laboratories, Hercules, CA, United States).

### Immunoblot Analysis

We extracted proteins from A549 cells using a lysis buffer (RIPA buffer: 100 mM phenylmethylsulfonyl fluoride, 10 mg/ml aprotinin, 1 M dithiothreitol). Thereafter, lysates were boiled for 10 min in sample-loading buffer (XT sample buffer 4× and XT reducing agent 20× mix; Bio-Rad). Proteins were separated by electrophoresis using Criterion TGX precast gels (4–15%) (Bio-Rad) and transferred to PVDF membranes (Immobilon; Millipore) by electroblotting. Samples containing 20 μg of protein were used for electrophoresis. The bands were detected using Amersham ECL Prime western blotting detection reagent (GE Healthcare, Chicago, IL, United States) and a luminescence image analyzer (LAS-4000IR; Fuji Film, Tokyo, Japan). Band intensities were quantified using ImageJ (National Institutes of Health, Bethesda, MD, United States).

### Animals

C57BL/6J mice (12- to 15-weeks old) were purchased from Charles River Laboratories Japan, Inc. (Kanagawa, Japan). The mice received a single transbronchial instillation of 2.0 mg/kg bleomycin on Day 0 to establish the pulmonary fibrosis model. Three times weekly from Day 7 to Day 21, 100 mg/kg of DFO or Hank’s Balanced Salt Solution (17461-05; Nacalai Tesque, Kyoto, Japan) was administered transbronchially. Thereafter, the murine lungs were dissected and embedded in paraffin, and the sections were stained with hematoxylin and eosin, and Masson’s trichrome. Fibrotic changes were evaluated using the Ashcroft score (Ashcroft et al., 1988). The hydroxyproline contents of the bleomycin-treated lungs were measured using a hydroxyproline colorimetric assay kit (K555-100; BioVision Inc.). Bronchoalveolar lavage (BAL) was performed with saline (1 ml) using a soft cannula. After the cell numbers in the BAL fluid were counted, cells were stained with Diff-Quik (16920; Sysmex, Tokyo, Japan) for classification. This animal study was reviewed and approved by the Animal Care and Use Committee at Nihon University School of Medicine.

### Data and Statistical Analysis

All data are presented as the mean ± standard error of mean (SEM). Statistical significance was analyzed using SPSS Statistics (v.21.0; IBM, Tokyo, Japan). Comparisons between the two independent groups were evaluated using the Student’s *t*-test. Two-way analysis of variance was conducted for experiments requiring multigroup comparisons. After the test, significant differences were evaluated using a multiple-comparison method (i.e., Newman–Keuls method). A *p* < 0.05 was considered significant for all analyses. Graphs were generated using GraphPad Prism software (v.6.0; GraphPad Software, Inc., La Jolla, CA, United States).

## Results

### Iron Chelation and a Ferroptosis-Specific Inhibitor Reduce Cigarette Smoke Extract-Induced Pulmonary Epithelial Cell Death

**FIGURE 1:**
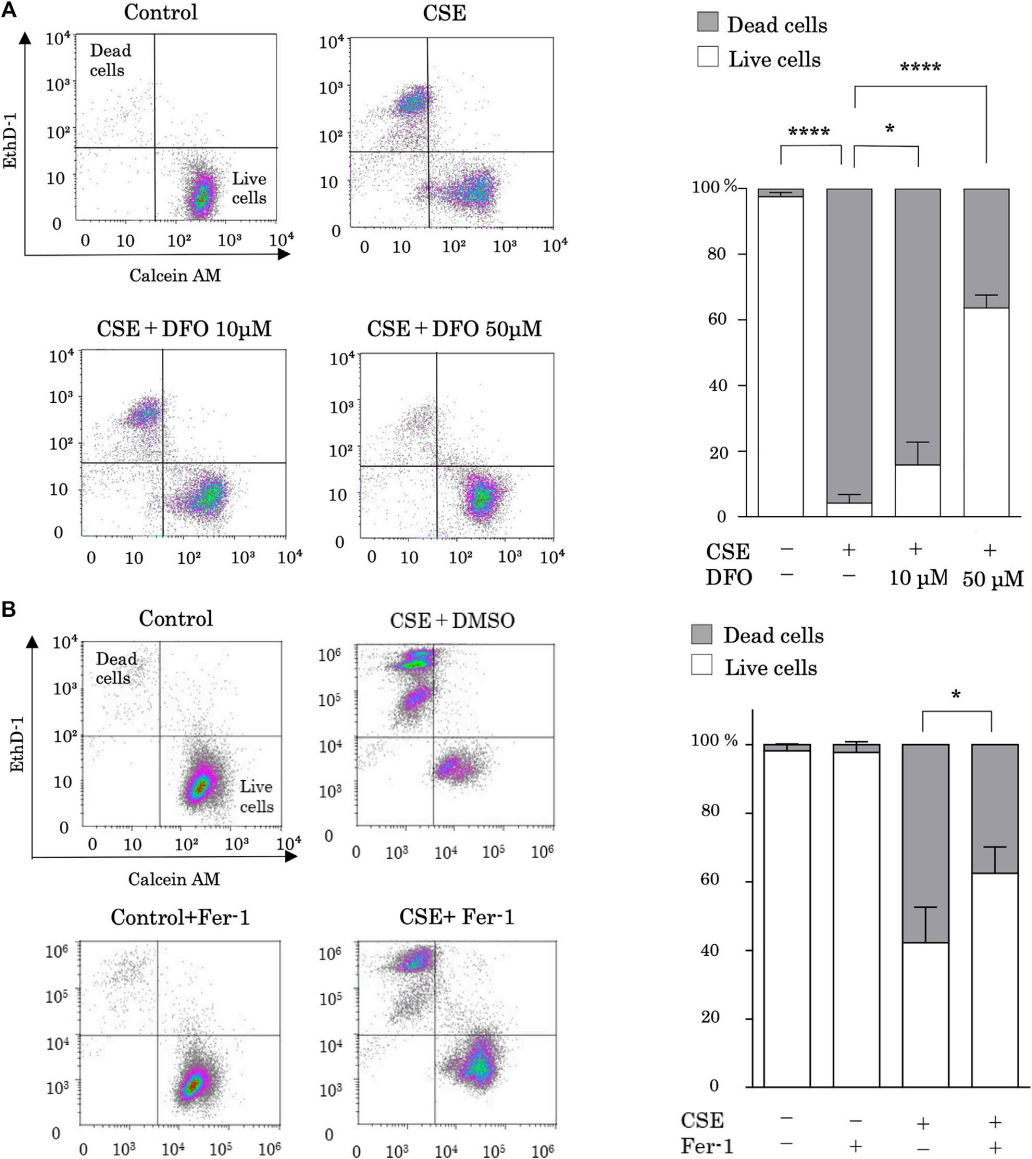
Iron chelation and a ferroptosis specific inhibitor reduced cigarette smoke extract (CSE)-induced pulmonary epithelial cell death. **(A)** BEAS-2B cells were incubated for 1 h with deferoxamine (DFO) (10 and 50 µM) or with a vehicle (sterile water) and treated with 25% CSE for 24 h **(B)** BEAS-2B cells were incubated for 1 h with ferrostatin-1 (4 µM) or vehicle (DMSO) and treated with 25% CSE for 24 h. Cell death was determined using calcein AM/EthD-1 flow cytometry. The x-axes show calcein-AM staining data, and the y-axes show EthD-1 staining data. Data are representative of five experiments. The percentages of live and dead cells are presented as bar graphs. All data represent the mean ± SEM. *p < 0.05 and ****p < 0.0001, based on two-way ANOVA with the Newman–Keuls *post-hoc* test.

Alveolar epithelium damage due to CS is the first stage of pulmonary fibrosis. To investigate the role of iron metabolism in CS-induced pulmonary epithelial cell death, we evaluated CSE-stimulated cell death in the presence of the iron chelator DFO. Previous studies have shown that CSE increased free iron concentration in BEAS-2B cells (Yoshida et al., 2019), and DFO treatment decreased intracellular labile iron (Bajbouj et al., 2018). Treatment with 25% CES for 24 h sufficiently induced pulmonary epithelial cell death (Figure 1A). CSE-induced cell death was effectively reduced by DFO treatment in a dose-dependent manner (Figure 1A). These data suggest that iron can regulate CSE-induced pulmonary epithelial cell death.

We then assessed whether ferroptosis is the major mechanism of CSE-induced cell death by using ferrostatin-1, a specific inhibitor of ferroptosis. Notably, treatment with ferrostatin-1 reduced CSE-induced cell death (Figure 1B).

### Iron Chelation Reduces Cigarette Smoke Extract-Induced Lipid Peroxidation in Pulmonary Epithelial Cells

**FIGURE 2:**
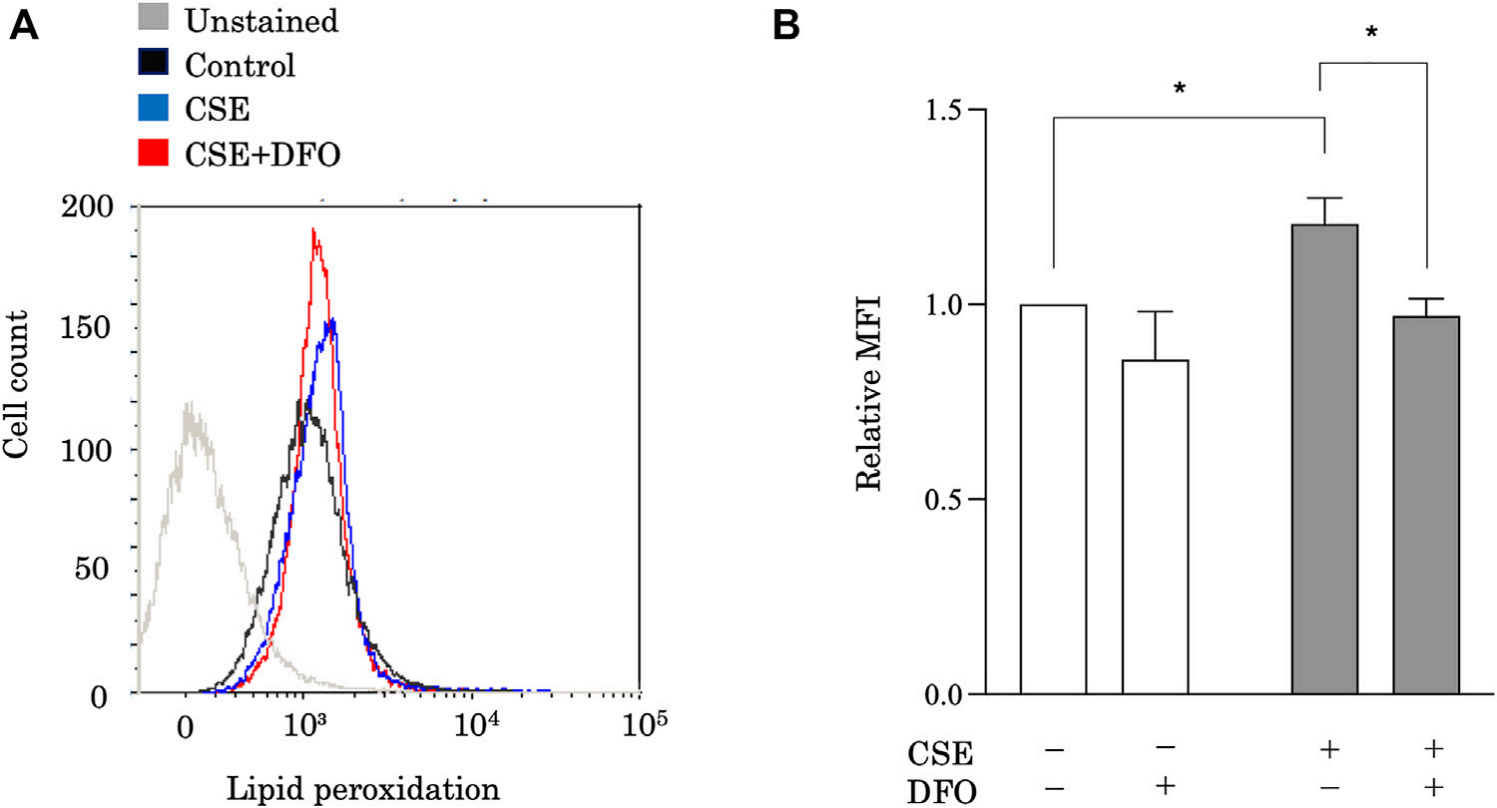
Iron chelation reduced CSE-induced lipid peroxidation in pulmonary epithelial cells. Flow cytometry of BEAS-2B cells left unstained or labeled with the redox-sensitive lipophilic dye BODIPY 581/591. BEAS-2B cells were incubated for 1 h with DFO (50 μM) and treated with 25% CSE for 24 h. The experiment was conducted three times independently under the same conditions. All data represent the mean ± SEM. *p < 0.05 based on two-way ANOVA with the Newman–Keuls *post-hoc* test. MFI, mean fluorescence intensity.

Ferroptosis is characterized by lethal levels of lipid peroxidation (Dixon et al., 2012). To study the effect of iron metabolism on lipid peroxidation, we treated BEAS-2B cells with CSE in the presence of the iron chelator DFO and evaluated lipid peroxidation using the redox-sensitive lipophilic dye BODIPY 581/591. BODIPY staining showed significant lipid peroxidation in response to CSE exposure (Figure 2). Moreover, we found that DFO treatment restored CSE-induced lipid peroxidation in BEAS-2B cells (Figure 2). Altogether, these findings implied that iron metabolism regulated CSE-induced pulmonary epithelial cell death through ferroptosis.

### Iron Chelation Reduces Cigarette Smoke Extract-Induced Mitochondrial Dysfunction in Pulmonary Epithelial Cells

**FIGURE 3:**
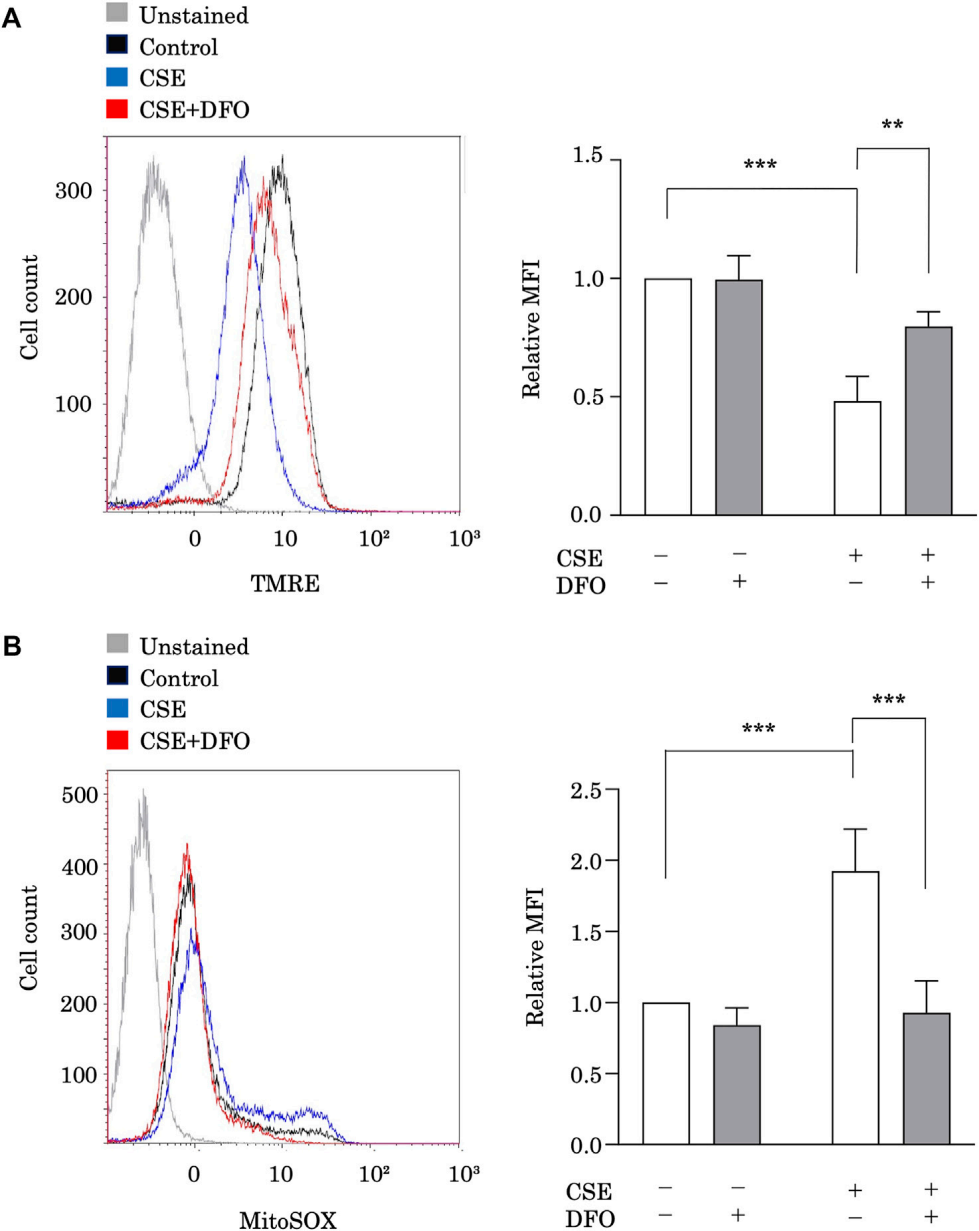
Iron chelation reduced CSE-induced mitochondrial dysfunction in pulmonary epithelial cells. **(A)** Flow cytometry of BEAS-2B cells left unstained or labeled with tetramethylrhodamine ethyl ester (TMRE). BEAS-2B cells were incubated for 1 h with deferoxamine (DFO) (100 μM) and treated with 5% CSE for 6 h. The experiment was conducted three times independently under the same conditions. **(B)** Flow cytometry of BEAS-2B cells labeled with MitoSOX. Cells were incubated for 1 h with DFO (100 μM) and then treated with 5% CSE for 6 h. The experiment was conducted three times independently under the same conditions. All data represent the mean ± SEM. **p < 0.01 and ***p < 0.001, based on two-way ANOVA with the Newman–Keuls *post-hoc* test. MFI, mean fluorescence intensity.

To study the effect of iron metabolism on mitochondrial integrity, we treated BEAS-2B cells with CSE in the presence of the iron chelator DFO and evaluated the functional mitochondrial pool using tetramethylrhodamine ethyl ester, a fluorescent probe sensitive to the mitochondrial membrane potential (ΔΨm) (Figure 3A). The functional mitochondrial pool was assessed after 6 h of CSE exposure, during which time the epithelial cells remained viable (data not shown). CSE treatment markedly decreased the number of functional mitochondria in BEAS-2B cells (Figure 3A). Interestingly, we found that DFO treatment restored the CSE-induced decline in ΔΨm (Figure 3A). Furthermore, we assessed mtROS production in BEAS-2B cells, as detected using MitoSOX, a fluorescent indicator of mitochondrial superoxide anion radical production (Figure 3B), and observed that CSE treatment predominately increased mtROS production in BEAS-2B cells (Figure 3B), and that treating these cells with DFO effectively reduced mtROS production (Figure 3B). These results suggested that the iron chelator preserved mitochondrial function and suppressed mtROS production in the CSE toxicity model.

### Iron Chelation and the Preservation of Mitochondrial Function Reduces TGF-β-Induced Epithelial-Mesenchymal Transformation

**FIGURE 4:**
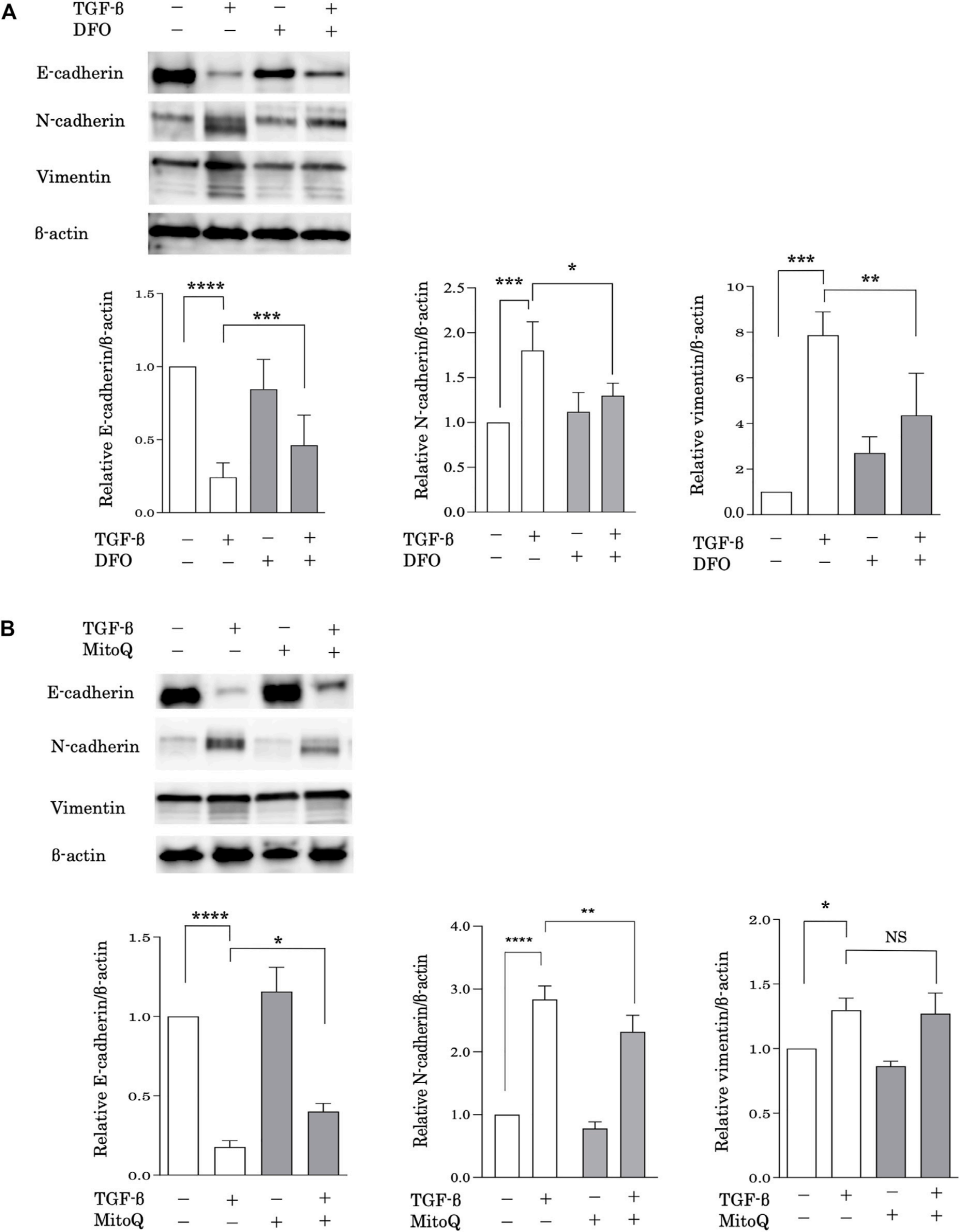
Iron chelation and the preservation of mitochondrial function reduced TGF-β-induced EMT. Immunoblot analysis for E-cadherin, N-cadherin and vimentin. **(A)** A549 cells were incubated for 1 h with deferoxamine (DFO) (10 μM) or vehicle and treated with TGF-β (10 ng/ml) for 72 h **(B)** A549 cells were incubated for 1 h with MitoQ (100 nM) or vehicle and then treated with TGF-β (10 ng/ml) for 72 h β-actin served as the standard. E-cadherin, N-cadherin, and vimentin expression was assessed by densitometric analysis of the immunoblots. Band intensities were normalized to that of β-actin. Data are representative of three experiments. All data are presented as the mean ± SEM. *p < 0.05, **p < 0.01, ***p < 0.001, and ****p < 0.0001, based on two-way ANOVA with the Newman–Keuls *post-hoc* test. NS, not significant.

EMT is considered a possible mechanism of pulmonary fibrosis after epithelial injury and during the healing process (Chapman, 2011). To evaluate the relationship between iron metabolism and EMT, alveolar epithelial A549 cells were pretreated with DFO after stimulation with transforming growth factor (TGF)-β. EMT was assessed based on the expression of E-cadherin, an epithelial cell marker, and N-cadherin and vimentin, mesenchymal cell markers, according to western blot analysis. Stimulation with TGF-β significantly decreased E-cadherin expression and increased N-cadherin and vimentin expression (Figure 4A), suggesting that TGF-β induced EMT. Remarkably, DFO treatment predominantly reduced TGF-β-induced E-cadherin downregulation and N-cadherin and vimentin upregulation (Figure 4A), suggesting that iron metabolism regulated TGF-β-induced EMT. We then employed MitoQ, a mitochondria-targeted antioxidant, to examine the involvement of mitochondrial dysfunction in EMT. Although TGF-β-induced vimentin upregulation was not reduced following MitoQ treatment, treatment with MitoQ predominantly reduced TGF-β-induced E-cadherin downregulation and N-cadherin upregulation (Figure 4B). These data indicated that mitochondrial dysfunction influenced TGF-β-induced EMT.

### Iron Chelation Reduces Cigarette Smoke Extract-Induced Mitochondrial DNA Release From Pulmonary Epithelial Cells

**FIGURE 5:**
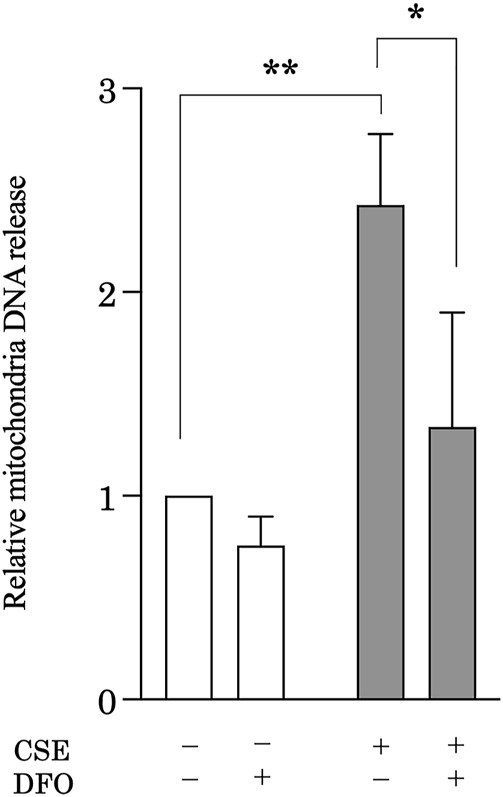
Iron chelation reduced CSE-induced mitochondrial DNA (mtDNA) release from pulmonary epithelial cells. BEAS-2B cells were treated with 5% CSE for 6 h, and the amount of mtDNA in cell supernatants was quantified by using quantitative real-time polymerase chain reaction. The y-axis shows the relative amount of mtDNA compared to that of the control. BEAS-2B cells were incubated for 1 h with deferoxamine (DFO) (50 μM) or vehicle and then treated with 5% CSE for 6 h. The experiment was conducted six times independently under the same conditions. All data are presented as the mean ± SEM. *p < 0.05 and **p < 0.01, based on two-way ANOVA with the Newman–Keuls *post-hoc* test.

CS induces mitochondrial iron overload, leading to mitochondrial dysfunction (Cloonan et al., 2016), which is associated with leakage of mtDNA (Riley and Tait, 2020). Accumulating evidence suggests that mtDNA acts as a damage-associated molecular pattern that can drive molecular processes that lead to inflammatory responses (Zhang et al., 2010a). Investigators recently reported that cellular mtDNA levels are decreased (Pyle et al., 2010; Ryu et al., 2017) and circulating cell-free mtDNA levels increased in response to stimuli, such as IPF (Ryu et al., 2017), trauma (Zhang et al., 2010a), and infection (Cossarizza et al., 2011; Sursal et al., 2013). Moreover, extracellular mtDNA levels can be predictive of mortality in IPF (Ryu et al., 2017) and intensive care unit patients (Nakahira et al., 2013). These observations led us to hypothesize that iron metabolism mediates CS-induced mtDNA release in pulmonary epithelial cells during the development of pulmonary fibrosis. To evaluate extracellular mtDNA, the amount of mtDNA in cell culture solution obtained from CSE-treated BEAS-2B cells was quantified using quantitative real-time polymerase chain reaction. Treatment with CSE significantly increased the amount of mtDNA in the cell culture supernatant as compared with that under control conditions, whereas DFO treatment effectively reduced CSE-induced mtDNA release into cell culture supernatant (Figure 5). These results suggested that iron metabolism regulated CSE-induced extracellular release of mtDNA, indicating the regulation of inflammatory responses through iron metabolism in pulmonary fibrosis.

### Transbronchial Iron Chelation Ameliorated Bleomycin-Induced Pulmonary Fibrosis

**FIGURE 6:**
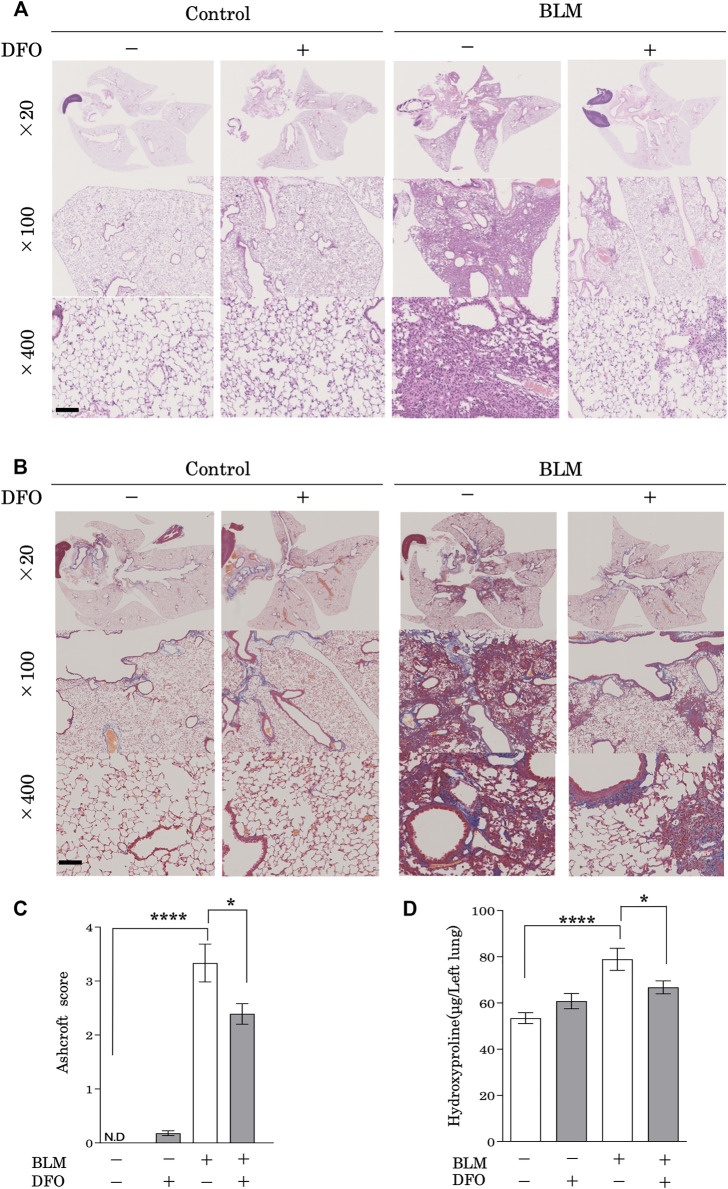
Transbronchial iron chelation ameliorated bleomycin-induced pulmonary fibrosis. Mice received a single transbronchial instillation of 2.0 mg/kg bleomycin (BLM) on Day 0. Thereafter, 100 mg/kg deferoxamine (DFO) or Hanks’ Balanced Salt Solution was administered transbronchially three times weekly from Day 7 to 21. On Day 28, the mice were euthanized, and their lungs were collected. Lung sections stained with **(A)** hematoxylin and eosin and **(B)** Masson’s trichrome. Scale bar: 100 μm. **(C)** Fibrotic changes in the lungs were histopathologically quantified based on a numerical fibrotic score (Ashcroft score). Six mice were used per group. Ten fields of view per mouse were randomly selected and examined. **(D)** Lungs were homogenized in distilled water, and the hydroxyproline contents were measured using the hydroxyproline assay. Ten mice were used per group. Data are presented as the mean ± SEM. *p < 0.05, ****p < 0.0001, based on two-way ANOVA with the Newman–Keuls *post-hoc* test. ND, not detected.

To examine the physiological function of iron metabolism in the pathological processes associated with pulmonary fibrosis, we used a mouse model of bleomycin-induced pulmonary fibrosis. Mice treated with bleomycin had damaged alveolar structures along with significantly increased interstitial thickening as compared with that in the control group (Figure 6A). Notably, a transbronchial injection of DFO ameliorated this bleomycin-induced interstitial thickening and destruction of lung alveolar structures (Figure 6A). Although lung collagen content was increased in bleomycin-treated mice as revealed by Masson’s trichrome staining, transbronchial injection of DFO ameliorated bleomycin-induced increases in lung collagen content (Figure 6B). Next, we evaluated fibrosis by measuring the Ashcroft histopathology score and confirmed significant increases in this score in bleomycin-treated mice (Figure 6C), with DFO injection reducing bleomycin-induced fibrosis according to the Ashcroft score (Figure 6C). The collagen content of the lungs, as quantified by determining hydroxyproline concentration, confirmed the successful induction of fibrosis by bleomycin (Figure 6D), whereas transbronchial injection of DFO ameliorated the bleomycin-induced increase in lung collagen content (Figure 6D). Additionally, we assessed the oral administration and intraperitoneal injection of DFO; however, these routes did not ameliorate the bleomycin-induced interstitial thickening or destruction of the lung alveolar structure (Supplementary Figures S1A,B). These data indicated that transbronchial intervention but not oral or intraperitoneal intervention of iron metabolism regulated lung fibrosis *in vivo*.

### Transbronchial Iron Chelation Reduced Bleomycin-Induced Lymphocyte Migration to the Murine Lung

**FIGURE 7:**
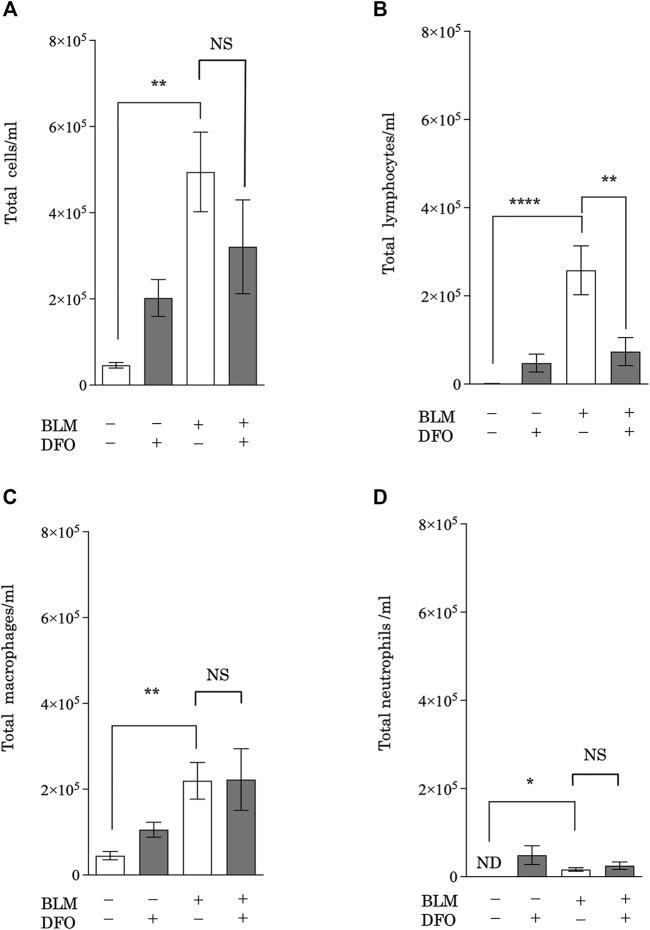
Transbronchial iron chelation reduced bleomycin-induced leukocyte migration in murine lungs. Quantification of changes in the total number of **(A)** BAL fluid cells, **(B)** lymphocytes, **(C)** macrophages, and **(D)** neutrophils. The number of cells was measured using an automatic cell counter. The ratio of lymphocytes, macrophages, and neutrophils were visually measured, and the number of these cells was calculated from the total number of cells. **p < 0.01 and ****p < 0.0001, based on two-way ANOVA with the Newman–Keuls *post-hoc* test. ND, not detected; NS, not significant.

After CS exposure, injured pulmonary epithelial cells release mediators that recruit leukocytes (Arnson et al., 2010), which then release profibrotic cytokines, such as interleukin (IL)-1β, tumor necrosis factor-α, IL-13, and TGF-β (Wynn, 2011). Therefore, leukocyte infiltration may be a critical event in pulmonary fibrosis. To assess whether iron metabolism regulates pulmonary inflammation, we analyzed BAL fluid from mice with bleomycin-induced pulmonary fibrosis and treated with or without DFO. Mice treated with bleomycin had higher total cell, lymphocyte, macrophage, and neutrophil counts in their BAL fluid (Figures 7A–D). Interestingly, DFO treatment significantly reduced the bleomycin-induced increase in lymphocyte number in the BAL fluid but did not reduce the numbers of total cells, macrophages, and neutrophils (Figures 7A–D). These results suggested that iron metabolism regulated bleomycin-induced lymphocyte migration but not macrophage and neutrophil migration in murine lungs.

## Discussion

An accumulating body of evidence suggests that iron metabolism is important in pulmonary diseases (Ghio et al., 2008; Cloonan et al., 2016; Cloonan et al., 2017). CS, a major risk factor for pulmonary fibrosis in humans, likely alters iron homeostasis in the lungs (Mussalo-Rauhamaa et al., 1986) and has been shown to notably induce mitochondrial iron loading in the lungs of mice (Cloonan et al., 2016). Extracellular iron is transported to mitochondria, where it is employed to synthesize cofactors essential to the functioning of enzymes (e.g., those involved in oxidation-reduction reactions, DNA synthesis, and a variety of other cellular processes). However, iron overload causes the Fenton reaction, which produces hydroxyl radicals at the cellular level and induces cell injury, cell death, fibrosis, and inflammation (Bogdan et al., 2016). Given that mitochondria are important generators of ROS, the coexistence of iron and ROS in the secluded mitochondrial space makes this organelle particularly prone to oxidative damage (Mena et al., 2015). In this study, we aimed to determine whether iron metabolism and mitochondrial dysfunction are involved in the pathogenesis of pulmonary fibrosis and revealed that the administration of the iron chelator DFO reduced CS-induced pulmonary epithelial cell death, mitochondrial dysfunction, and mtDNA release from pulmonary epithelial cells. Interestingly, treatment with a specific inhibitor of ferroptosis, ferrostatin-1, reduced CSE-induced pulmonary epithelial cell death. TGF-β-induced EMT of cultured pulmonary epithelial cells was also reduced with DFO treatment. In addition, the preservation of mitochondrial function reduced TGF-β-induced EMT, and transbronchial iron chelation ameliorated bleomycin-induced pulmonary fibrosis and leukocyte migration in a murine model. Taken together, these results suggest a pro-pathogenic role of iron metabolism and mitochondrial dysfunction in CS-induced pulmonary fibrosis.

In this study, we found that treatment with the iron chelator DFO effectively reduced CSE-induced cell death. We had previously observed that pulmonary epithelial cell mitochondrial damage due to CS is involved in necroptosis (Mizumura et al., 2014), while recent studies suggest the existence of a unique iron-dependent form of nonapoptotic cell death, termed “ferroptosis,” which is characterized by the accumulation of lipid peroxides (Dixon et al., 2012). Therefore, we assessed whether ferroptosis is the main mechanism of iron mediated CSE-induced cell death. Treatment with a specific inhibitor of ferroptosis, ferrostatin-1, significantly reduced CSE-induced cell death. We also revealed that treatment with DFO restored the CSE-induced decline in ΔΨm and effectively inhibited CSE-induced production of mtROS in BEAS-2B cells. Consistent with our results, recent studies have revealed that mitochondria play a crucial role in ferroptosis (Gao et al., 2019; Tadokoro et al., 2020). Inhibition of the mitochondrial tricarboxylic acid cycle or electron transfer chain alleviated ΔΨm hyperpolarization, lipid peroxide accumulation, and ferroptosis (Gao et al., 2019). Our data, along with those of previous reports, suggest that iron metabolism regulates CSE-induced cell death through the mitochondria-ferroptosis pathway.

EMT during the healing process and following epithelial injury is a possible mechanism of pulmonary fibrosis (Chapman, 2011). In injured lung tissues, epithelial cells lose their tight junctions and are transdifferentiated to mesenchymal cells via EMT (Willis and Borok, 2007). Thereafter, fibroblasts are activated and differentiate into myofibroblasts, which is a process called fibroblast-myofibroblast transition (FMT). Myofibroblasts are specialized contractile cells with a higher profibrotic potential than fibroblasts (King et al., 2011). FMT is described as a multifactorial process that involves enhanced force generation with enlarged focal adhesions and incorporation of α-smooth muscle actin (α-SMA) into stress fibers (Hinz et al., 2012; Guo et al., 2014). TGF-β is a critical cytokine for EMT and FMT (Willis and Borok, 2007). Moreover, increased contraction of extracellular matrix fibers can uncage TGF-β from its latent complex and release it into the medium (Klingberg et al., 2014). Several studies (Willis et al., 2005; Larsson et al., 2008; Marmai et al., 2011) have indicated that the alveolar epithelium may contribute to the expansion of fibroblasts and myofibroblasts in injured lung areas through EMT and FMT, although the precise mechanisms involved remain to be elucidated. In this study, we found that iron chelation reduced TGF-β-induced EMT. Furthermore, we revealed that iron chelation effectively reduced the production of mitochondrial ROS in CSE-treated epithelial cells. ROS play an important role in TGF-β-induced EMT in renal tubular epithelial cells through the activation of mitogen-activated protein kinases (Rhyu et al., 2005). In addition, ROS are involved in the mediation of matrix metalloproteinase 3-induced EMT by stimulating the expression of the transcription factor Snail (Radisky et al., 2005). Consistent with these observations, we found that the preservation of mitochondrial function reduced TGF-β-induced EMT. These findings indicate that iron metabolism may regulate TGF-β-induced EMT through ROS production in the alveolar epithelium. Most studies currently acknowledge that EMT occurs in IPF to some extent (Kim et al., 2009; Chilosi et al., 2017; Yamaguchi et al., 2017; Yao et al., 2019); however, it must be noted that the *in vivo* occurrence of EMT is controversial. Lineage-tracing studies report conflicting results that either support or deny the pathogenetic role of EMT in lung fibrosis (Rock et al., 2011). Therefore, the present findings that iron chelation and the preservation of mitochondrial function reduced TGF-β-induced EMT should be carefully evaluated in the context of IPF pathogenesis. Considering the most widely recognized pathogenetic mechanisms of IPF, it seems appropriate to consider the dysregulated EMT process as a contrary response relative to the proper EMT observed during lung repair and regeneration in healthy subjects (Salton et al., 2019). Further studies are needed to better elucidate the mechanisms and relevance of EMT in IPF *in vivo*.

Studies suggest that mitochondrial damage is involved in intracellular mechanisms and intercellular signaling due to the release of mitochondrial damage-associated molecular patterns (Nakahira et al., 2015). The release of mtDNA into blood is increased in several disease models (Zhang et al., 2010b; Duprez et al., 2011; Evdokimovsky et al., 2011), and mtDNA regulates inflammation through the NALP3 inflammasome (Nakahira et al., 2011; Zhou et al., 2011). Recent research indicates that mtDNA concentrations are increased in BAL fluid and in the plasma of patients with IPF; these concentrations display robust associations with disease progression and reduced event-free survival (Ryu et al., 2017). However, the precise mechanisms by which pulmonary cells secrete mtDNA remain obscure. We found that treating pulmonary epithelial cells with CSE significantly increased the amount of mtDNA in the cell culture supernatant. Notably, CSE-induced mtDNA release was effectively reduced by DFO treatment. Our finding provides supporting evidence that iron metabolism mediates intercellular signaling by regulating mtDNA release.

Consistent with the pathogenic role of iron metabolism in CS-dependent responses, we demonstrated that transbronchial iron chelation, but not oral or intraperitoneal iron chelation, ameliorated bleomycin-induced pulmonary fibrosis in a murine model. Furthermore, we showed that transbronchial iron chelation reduced bleomycin-induced leukocyte migration, but not macrophage and neutrophil migration, in murine lungs. The presence of T lymphocytes within the lung tissue (Kradin et al., 1986; Parra et al., 2007; Papiris et al., 2015) and BAL fluid (Fireman et al., 1998; Pignatti et al., 2006) of patients with IPF has been observed consistently. However, it appears unlikely that T lymphocytic infiltration *per se* is the central driving force for fibrotic processes in most cases of pulmonary fibrosis (Helene et al., 1999; Luzina et al., 2008). Conversely, previous studies suggest that T lymphocytes may modulate the inflammatory and healing responses in the lungs in a profibrotic or antifibrotic manner, depending on their phenotype (Luzina et al., 2008; Desai et al., 2018). Therefore, our finding that DFO reduces bleomycin-induced lymphocyte migration in the murine lung provides supporting evidence that iron modifies lung fibrosis. However, further investigation of lymphocyte migration and iron metabolism to focus on the phenotype of lymphocytes and fibrotic stage (i.e., profibrotic or antifibrotic stage) is needed for a better understanding of the pathogenesis of pulmonary fibrosis.

Although a recent study revealed that ferroptosis is involved in the pathogenesis of chronic obstructive pulmonary disease (Yoshida et al., 2019), the role of ferroptosis in pulmonary fibrosis remains obscure. In this study, we could not conclusively evaluate the involvement of iron metabolism-regulated lymphocyte migration and ferroptosis in the pathogenesis of human pulmonary fibrosis. Nevertheless, our finding that transbronchial iron chelation ameliorated bleomycin-induced pulmonary fibrosis and leukocyte migration in a murine model provides supporting evidence for the role of iron metabolism in the pathogenic process of pulmonary fibrosis.

In conclusion, we found that iron chelation reduces CSE-induced cell death, mitochondrial dysfunction, and EMT in pulmonary epithelial cells. Our data demonstrated that transbronchial iron chelation ameliorated pulmonary fibrosis and leukocyte migration *in vivo*, which supports our hypothesis that iron metabolism contributes to the pathogenesis of pulmonary fibrosis. Our study provides a mechanistic explanation for how CS may regulate pulmonary fibrosis through iron metabolism via mitochondrial dysfunction. Strategies targeting this pathway, such as DFO treatment, may lead to novel therapies for pulmonary fibrosis.

## Data Availability

The original contributions presented in the study are included in the article/Supplementary Material, further inquiries can be directed to the corresponding author.

## Abbreviations

ANOVA, analysis of variance; BAL, bronchoalveolar lavage; CS, cigarette smoke; CSE, cigarette smoke extract; DFO, deferoxamine; EMT, epithelial-mesenchymal transformation; FMT, fibroblast-myofibroblast transition; IPF, idiopathic pulmonary fibrosis; mtDNA, mitochondrial deoxyribonucleic acid; mtROS, mitochondrial reactive oxygen species; ROS, reactive oxygen species; SEM, standard error of the mean; TGF-β, transforming growth factor beta.
