# Supplementary figures and images for: Iron-Dependent Mitochondrial Dysfunction Contributes to the Pathogenesis of Pulmonary Fibrosis
Source: Front Pharmacol. 2022 Jan 4;12:643980. doi: 10.3389/fphar.2021.643980 (PMC8765595; doi:10.3389/fphar.2021.643980)

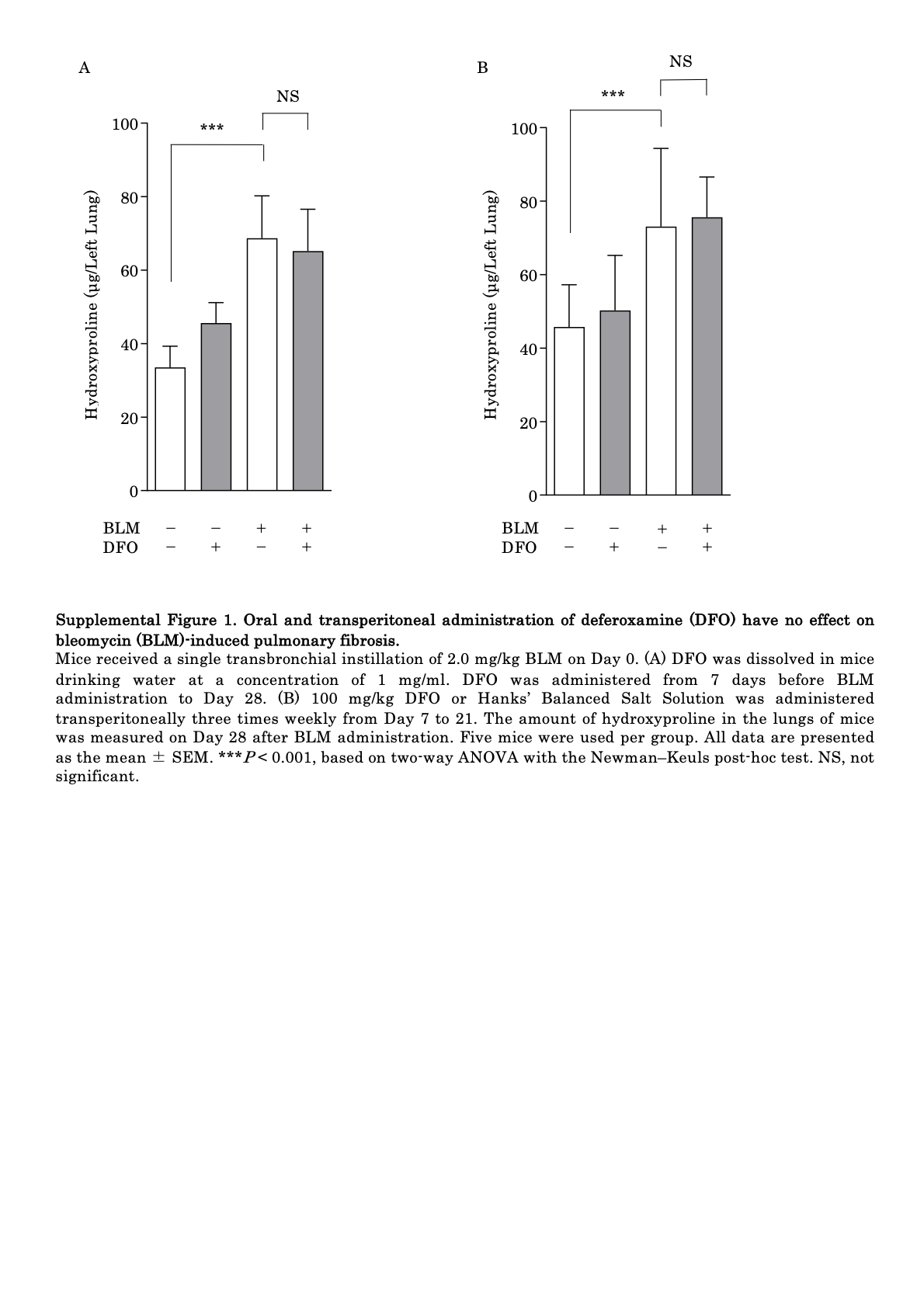

Supplement: Supplementary file 1 [file Image1.TIFF]
